# Prevalence of and risk factors for overweight among adolescents of a sub-metropolitan city of Nepal

**DOI:** 10.1371/journal.pone.0270777

**Published:** 2023-03-06

**Authors:** Elina Khatri, Kedar Baral, Amit Arjyal, Rajesh Kumar Yadav, Sushila Baral

**Affiliations:** 1 School of Public Health, Patan Academy of Health Sciences, Lalitpur, Nepal; 2 Research Section, Nepal Health Research Council (NHRC), Kathmandu, Nepal; 3 Department of Public Health, School of Health and Allied Sciences, Pokhara University, Pokhara, Nepal; 4 Department of Research, Health Research Together Initiative (HeaRT: Initiative), Kathmandu, Nepal; 5 Department of Health Training, Provincial Health Training Centre, Pokhara, Nepal; Al-Ahliyya Amman University, JORDAN

## Abstract

**Background:**

Overweight is a global public health problem with increasing trend especially in middle to lower socioeconomic country like Nepal. The nutritional status of adolescents being shaped by socio-cultural, environmental, and economic factors has also been impacted by their food habits and level of physical activity. The current nutritional shift and rapid urbanization had emerged overweight as an additional burden for consistently prevalent undernutrition issues. So, the study aimed to identify the prevalence of and risk factors for overweight among school adolescents.

**Methods:**

A cross-sectional analytical study was carried out among random sample of 279 adolescents from nine schools of a Sub-metropolitan city of Nepal. The anthropometric measurement of the height and the weight were taken as per the standard. The odds ratio with a 95% CI was calculated and a p-value of ≤0.05 was considered as cut off for statistical significance by fitting into the final multivariable logistic regression.

**Results:**

The overall prevalence of overweight was obtained as 9.31% (95% CI: 6.40–13.3). The early aged adolescents were more overweight than compared to middle-aged adolescents (AOR: 0.27, CI: 0.028–2.67) and late adolescents (AOR: 0.66, CI: 0.068–6.44) respectively. Similarly, adolescents residing in rural areas had 0.35 (AOR = 0.33, CI: 0.030–3.71) odds of being overweight compared to their counterparts. Adolescents with sedentary behavior were about 4 times (AOR = 3.51, CI: 0.79–15.54) more likely of being overweight than their counterparts.

**Conclusion:**

Overweight among adolescents residing in urban areas has emerged as an alarming issue due to their unhealthy lifestyle habits. It is therefore pertinent to emphasize adolescents to maintain healthy weight status through health food habits and physical activity.

## Introduction

Overweight is the condition characterized by an abnormal and extreme accumulation of fat which can worsen bodily functions [1]. It is one of the major risk factors for most chronic illness related to diet and body mass index [2] and is considered a multifactorial chronic conditions that is affected by genetic, metabolic, behavioral, environmental, cultural and socioeconomic factors [3]. Adolescence, being a crucial stage for experimentation and acceptance of new behavioral and lifestyle choices, is detrimental to their nutritional status [4]. The nutritional status of adolescents being shaped by socio-cultural, environmental, and economic factors has also been impacted by their food habits and level of physical activity [5]. Although obesity and overweight have become pandemic even in the developing world, it has been a most neglected problem until quite recently [6]. However, the burden of overweight among adolescents has been increased in both developed and developing countries [5]. Globally, overweight has nearly been tripled since 1975 with more than 340 million children and adolescents aged 5–19 years being overweight in 2016 [1]. Approximately 170 million adolescents i.e. children under 18 years were overweight or obese in 2008 and it has been estimated that around 30% of all children will be obese or overweight by 2030 [7]. Such conditions is termed as "New World Syndrome" as 40–50 million school-aged children worldwide are obese [3]. According to the World Health Organization (WHO), maximum percentage of overweight children is in developing countries with a higher rate of increment as compared to the developed world. A comparative study conducted in developing countries had reported the higher prevalence rates of obesity among adolescents in Asia [7].

The current shift in lifestyle and change in consumption patterns of high energy-dense diets which are rich in fat and calorie content and low intake of micronutrients such as vitamins and minerals could be the reasons towards overweight among adolescents [7]. In developing countries, the advancement in the accessibility of food and decrease in physical activity had been recognized as major contributing factors for increasing trend of overweight and other chronic metabolic diseases [8]. In addition to this, physical activity during adolescence stage is considered to have a positive impact on physiological and psychological growth and development which is continued to adulthood [9].

The prevalence and risk associated with overweight and obesity among adolescents in developing nations are increasing at a higher rate as compared to developed. However, the risk factors associated with overweight and obesity are not well understood in Nepal [10]. Nearly a quarter of population in Nepal are adolescents which cover about 23.45 percent of the total population [11]. The problem of overweight and obesity is also becoming public health challenge along with the consistent problem of undernutrition [12]. Although fewer studies done among adolescents of Nepal have reported the prevalence of overweight as 12.2% [10], 8.1% [13], and 1% [11], the risk factors related to the overweight studies has not been well explored. The unavailability of data with standard cut-off values and references has made it difficult for comparisons and trend analysis of overweight among adolescents worldwide [8]. This study aims to identify the prevalence of and associated risk factors of overweight among school adolescents of the sub-metropolitan city, Nepal.

## Materials and methods

### Study design

The cross-sectional analytical study was done among the adolescents from secondary schools of Hetauda Sub-Metropolitan City, Makwanpur district.

### Sample size

The study participants were selected randomly based on their enrollment in either government or private schools. The sample size was obtained as 272 using a single proportion formula considering the prevalence of overweight among higher secondary school adolescents as 12.2% [10] with 95% confidence interval at 5% margin of error, 10% of non-response rate and design effect as 1.5.

### Sampling techniques

Sampling was done by using multi-stage random sampling. The updated list of secondary schools was obtained from the concerned authorities. Since there was a significant difference in the number of students from government and private schools, based on proportional allocation 6 government and 3 private schools were selected through Probability Proportionate to Size (PPS) sampling. Finally, participants were selected in equal distribution using the Systematic Random Sampling (SRS) technique.

### Study variables

#### Adolescents

In this study, individuals in the age group of 10–19 years of age were considered as adolescents with adolescents in the age group of 10–13 years, 14–16 years, and 17–19 years being early, middle and late adolescents respectively.

#### Body Mass Index (BMI)

Based on 2007 WHO reference, BMI for age greater than 1 z scores were considered as overweight adolescents.

#### Lifestyle habits

The food habits was assessed by using food frequency questionnaire for consumptions pattern. Similarly, leisure time physical activity adolescents was assessed using WHO structured questionnaire. Adolescents were categorized as “Leisure Time Physical Activity” if they were engaged in any form of leisure time activities for more than 10 minutes in any day of a week and for those who did not do such activities were categorized as “No Leisure Time Physical Activity”.

#### Sedentary behavior

The total number of hours’ adolescents spent on sitting per day was considered for assessing sedentary behavior. The sitting hours above 6 hours was considered as adolescents having sedentary behavior.

#### Screen time

Excessive screen time was defined as watching television and playing video games or using any electronic devices for more than 2 hours per day [13].

### Study tools

Data was collected using a validated and structured self-administered tool for assessing adolescents’ socio-demographic characteristics, food habits, physical activity, and anthropometric measurements. Food habits was assessed using food frequency table for consumptions of vegetables, fruits, meat, fish, egg, fast food, junk foods and soft/sweetened drinks per week. The tool used in Adolescent Nutrition Survey, 2014 [11] was adopted to assess socio-demographic characteristics and food habits of adolescents. The food items specifically relevant to adolescents in current perspective were listed to assess the food habits of adolescents using Food frequency questionnaire. Likewise, physical activity of adolescents during leisure time for vigorous and moderate activities were assessed based on WHO STEPS surveillance for non-communicable diseases’ risk factors [14]. An electronic SECA digital weighing scale (UNICEF Electronic Scale) was used to measure the weight of all adolescents. Height was measured by using a standard height measuring board (Shorr-Board) in a standing position by following standard procedures.

### Data management

Data were coded and entered in Epi Info 7 and analyzed using STATA MP 13 software. However, the anthropometric assessment was done in WHO Anthro Plus Software V.1.0.4 to monitor the BMI status of adolescents. Data were expressed in frequency, percentage, mean and standard deviation. Bivariate and multivariate logistic regression analyses were conducted to determine the association between dependent and independent variables.

### Ethical approval

Ethical approval was obtained from the Institutional Review Committee (IRC) of Patan Academy of Health Sciences (PAHS). Approval from the municipal authorities and the school administration was also obtained. Adolescents were provided with an informed consent form prior the day of data collection to sign by their parents or guardians. Only those adolescents who brought the written informed consent form signed by their parents were included in the study. Participants were explained about the research detail, its significance, the benefit, and the harm. Participants were informed about their right to withdraw from the study at any time without giving any reason. The confidentiality of the research participants was maintained by providing separate identity number (ID) to each participant’s instead of their personal information during data collection. The anthropometric measurement was taken individually in separate room.

## Results

The socio-demographic information of school adolescents such as age, sex, place of residence, ethnicity, religion, parental education and employment status, school type, birth order, and physical environmental factors was assessed for descriptive analysis as well as understanding its association with overweight among adolescents.

Almost 56% of participants belong to middle adolescents followed by 29.39% in later adolescents with 14.70% in early adolescents group. More than half of study participants were female i.e. 56.63% with 43.37% as male adolescents. The majority of adolescents i.e. 90% reported as urban residents with 10% residing in rural areas. Also, in this study about two-third of school adolescents were from government. More than half i.e. 61.29% of school adolescents belong to the nuclear family. Likewise, more than one-fourth i.e. 29.03% of adolescent’s mothers were without any education followed by 22.22% of them educated up to secondary level and 17.92% and 17.20% educated through informal education and educated up to primary level respectively with few i.e. 9.68% educated up to higher secondary level. Similarly, 29.03% of adolescent’s father was educated up to secondary level. However, 12.19% of fathers were without education. More than half i.e. 55.20% of the adolescent’s mothers were found to be house makers followed by 24.37% of mothers engaged in agriculture as their occupation (Table 1).

**Table 1:** Demographic characteristics of adolescents (n = 279).

Variables	Frequency (n)	Percentage (%)
**Age (Completed years)**		
11–13 (early adolescents)	41	14.70
14–16 (middle adolescents)	156	55.91
17–19 (late adolescents)	82	29.39
**Sex**		
Male	121	43.37
Female	158	56.63
**Place of Residence**		
Urban	250	89.61
Rural	29	10.39
**Birth Order**		
First	117	41.94
Middle	70	25.09
Last	92	32.97
**Type of School**		
Government	186	66.67
Private	93	33.33
**Type of Family**		
Nuclear	171	61.29
Joint	108	38.71
**Mother’s Education Level**		
Illiterate	81	29.03
Informal education	50	17.92
Primary	48	17.20
Secondary	62	22.22
Higher secondary	27	9.68
University	11	3.94
**Father’s Education Level**		
Illiterate	34	12.19
Informal education	37	13.26
Primary	55	19.71
Secondary	81	29.03
Higher secondary	51	18.28
University	21	7.53
**Mother’s Employment**		
House maker	154	55.20
Agriculture	68	24.37
Labor	6	2.15
Government services	12	4.30
Private job	16	5.73
Business	18	6.45
Others	5	1.79
**Father’s Employment**		
Agriculture	58	20.79
Labor	25	8.96
Government services	29	10.39
Private job	46	16.49
Business	65	23.30
Foreign employment	44	15.77
Others	12	4.30

The mean weight for adolescents was found to be 48.91 kg (95% CI 47.83–49.99) with minimum and maximum weight as 29.3 kg and 95 kg respectively. The reported average height of adolescents was found to be 157.37 cm (95% CI 156.43–158.31) with minimum and the maximum height of adolescents as 130 cm and 179.5 cm. The Body Mass Index age score was computed for categorizing adolescents as overweight. The nutritional status of adolescents was done based on the BMI for age z scores. The mean BMI for age was computed as 19.72 kg/m^2^ with minimum and maximum as 13.74 kg/m^2^ and 36.14 kg/m^2^ respectively. Approximately 9.32% of adolescents were found to be overweight (i.e. heavy for their age) (Table 2).

**Table 2:** Anthropometric assessment of adolescents (n = 279).

Overweight	Frequency (n)	Percentage (%)	95% CI
Yes	26	9.32	6.40–13.36
No	253	90.68	86.63–93.59

### Factors associated with overweight among adolescents

The independent variables were separately assessed with overweight using bivariate regression to assess risk factors associated with overweight. The age of adolescents was found to be significantly associated with their overweight status. No significant association was found between religion, ethnicity and type of family of adolescents with their overweight status. The association of food habits with overweight was analyzed.

This finding showed the significant relationship between age of adolescents and overweight with middle-aged adolescents (14–16 years) having 0.24 (p = 0.005) odds of being overweight than that of early adolescents. While for late adolescents aged 17–19 years the odds of being overweight is 0.33 (p = 0.044) times compared to early adolescents. Similarly, the adolescents residing in urban areas were found to be more overweight as compared to rural adolescents. The adolescent’s with mother having university degree has 8.68 (p = 0.005) odds of being overweight as compared to illiterate mother. Sedentary behavior was found to be associated with overweight. The adolescents spending more than 6 hours per day in a sitting position were found to have 2.08 odds to be overweight than their counterparts. This analysis showed that skipping dinner is statistically associated with overweight which means this variable has an independent effect on overweight while controlling for other variables (Table 3).

**Table 3:** Factors associated with overweight.

Characteristics	Overweight	Unadjusted analysis		Adjusted analysis
	%	p value	OR (95% CI)	AOR (95% CI)
**Age (Years)**		0.02*		
11–13	21.95		1	1
14–16	6.41		0.24 (0.091–0.647)	0.27 (0.028–2.67)
17–19	8.54		0.33 (0.113–0.968)	0.66 (0.068–6.44)
**Place of Residence**		0.19		
Urban	10		1	1
Rural	3.45		0.32 (0.041–2.464)	0.33 (0.030–3.71)
**Birth Order**		0.05		
First	13.68		1	1
Second	8.57		0.59 (0.22–1.59)	0.13 (0.011–1.61)
Last	4.35		0.28 (0.03–0.092)	0.19 (0.026–1.34)*
**Mother’s Educational Level**		0.06		
No education	6.17		1	1
Informal education	6		0.97 (0.22–4.24)	0.44 (0.03–5.88)
Primary	5.25		1.01 (0.23–4.44)	0.27 (0.018–4.15)
Secondary	9.68		1.62 (0.47–5.60)	0.70 (0.068–7.14)
Higher secondary	18.52		3.45 (0.91–13.02)	5.73 (0.41–79.64)
University	36.36		8.68 (1.88–39.94)	0.80 (0.02–22.50)
**Father’s Employment**		0.07		
Agriculture	6.90		1	1
Government services	6.90		1 (0.17–5.80)	0.45 (0.021–9.45)
Private job	6.52		0.94 (0.19–4.43)	0.85 (0.06–11.39)
Business	10.77		1.62 (0.45–5.87)	0.93 (0.11–7.88)
Foreign employment	11.36		1.73 (0.43–6.86)	1.5 (0.13–17.10)
Others	41.67		9.64 (2.08–44.64)	12.0 (0.35–411.6)
**Frequency of skipping meals**		0.21		
Never	6.25		1	
1–3 times per week	12.71		2.18 (0.89–5.35)	1
More than 3 times per week	9.09		1.5 (0.37–5.99)	0.52 (0.080–3.46)
**Type of skipped meals**		0.12		
Breakfast	14.71		1	1
Lunch	5.88		0.36 (0.065–2.01)	1.59 (0.12–20.47)
Day Snacks	5.41		0.33 (0.05–1.83)	2.97 (0.20–44.12)
Dinner	19.57		1.41 (0.42–4.66)	12.6 (1.39–114.23)*
**Consumption of green leafy vegetables per week**		0.16		
Never	8.33		1	1
1–3 times per week	7.55		0.89 (0.10–7.40)	0.82 (0.05–12.86)
More than 3 times per week	16.36		2.15 (0.24–18.81)	0.88 (0.038–20.39)
**Sedentary behavior**		0.07		
<6 hrs	6.54		1	1
>6 hrs	12.70		2.08 (0.90–4.76)	3.51 (0.79–15.54)

Similarly, in final model, middle and later aged adolescents were found to have 0.27 and 0.66 odds of getting overweight compared to earlier adolescents keeping other variables constant. In addition to this, urban adolescents were found to be more overweight than their counterparts while controlling for other variables. Similarly, mothers educated up to higher secondary and university level were found to have 5.73 and 0.80 odds of getting overweight adolescents respectively (Table 3). Moreover, adolescents skipping meals more than three times per week were found to have 0.52 odds of being overweight compared to those whose who skip one to three times while controlling for other variables. In addition to this, adolescents skipping dinner were 12.62 times more likely to get overweight than those who skip breakfast with a significant result while controlling other variables. Adolescents having sedentary behavior (spending more than 6 hours a day in sitting/reclining position) were 3.51 times more likely to be overweight compared to their counterparts while controlling for other variables. However, the result is not statistically significant (Table 3).

## Discussion

This study was conducted to assess the prevalence of and risk factors associated with overweight among adolescents. The overall prevalence of overweight was obtained as 9.34% which is approximately similar to findings from other studies done in urban areas in Nepal. The current finding is similar to a study done in Kaski district which reported 8.1% prevalence of overweight and obesity among school adolescents with 5.8% and 2.3% prevalence of overweight and obesity respectively [13]. However, this finding is slightly lower than another study done among urban school adolescents in Lalitpur municipality which reported prevalence of overweight as 12.2% (95% CI 8.9 to 15.5) [10] which might be due to variations in study sites as Hetauda is still considered as periphery in comparison to Kathmandu valley where lifestyle habits are modernized in terms of food habits and physical inactivity. Similarly, this prevalence is higher as compared to another study done in Kaski district which stated overweight prevalence as 3.3% [15]. This finding stands in contrast with Adolescent Nutrition Survey, 2014 which found only 1% of adolescent as overweight [11].

Also, this study is supported by various other studies done in South Asian countries such as India, Pakistan and Bangladesh. The study done in Pakistan in 2012 reported prevalence of overweight as 8% among adolescents which is almost similar in this study context [16]. Similarly, two cross-sectional studies done in Bengal and Belgaum city of India found 6.75% [17] and 12% [18]. This difference might be due to larger economy, wealthier people and adoption of a sedentary lifestyle. This unequal distribution in the prevalence of overweight among school adolescents might be due to variation in adopting a sedentary lifestyle in different study settings.

The present study assessed the risk factors associated with overweight among adolescents. Sex and type of school found no significant association with overweight. In contrast to this, a study done in Lalitpur city found the significant relationship of overweight with adolescents being male and studying in private school [10] which might be due to least variation between types of school in study area as compared to urban areas. Similarly, the insignificant relationship of age, ethnicity, family type, and mothers’ occupation with overweight depicted in that study are also comparable to current study [13].

The study done by Piryani S et al. among urban adolescents of Kathmandu valley found consumption of fruits and vegetables four times or less per week associated with overweight [10] which is comparable to current study findings. Furthermore, another cross-sectional study done among adolescents in Kaski district reported having meals more than three times per day (p<0.001, OR = 14.06) and consuming vegetables more than three times per week (p<0.001, OR = 2.74) as an associated factor for overweight [13] which contradicts with current findings depicting no significant association of meal frequency and consumption of vegetables with overweight. The association of vegetable consumption for more than three times per week is comparable with the same study as adolescents consuming green leafy vegetables more than three times per week had 0.82 lesser odds of being overweight compared to those who never consumed. This might be due to over or under-reporting of adolescents as food consumption pattern was assessed by using a frequency-based questionnaire which can pose a threat of recall bias. This variation might also be due to variation in availability and accessibility of foods in study site, being the junction of foothills and Terai region. The other reasons could be due to variation in the lifestyle of people residing in peri-urban areas under the recently formed sub-metropolitan city area.

The study done by Acharya B et al. to identify the prevalence and factors associated with overweight among adolescents in Kaski district revealed that performing a vigorous activity and involving in passive activities during leisure are factors associated with overweight [13]. In contrast to this, present study did not find a significant association of overweight with vigorous physical activity. This study showed an association between overweight and sedentary behavior of participants i.e. spending more than 6 hours a day in a sitting position (p = 0.09, OR = 3.51). Similar findings have been obtained in a case-control study done in India which depicted engagement in sedentary behavior for more than 4 hours a day (OR = 2, p = 0.02) as a risk factor for overweight [19]. This study support the current findings which showed insignificant association of overweight with sex of adolescents and their level of physical activity.

This study has not found the significant association of overweight with their lifestyle habits as mentioned in literature which may be due to the cross-sectional nature of the study being unable to check association. Also, the lifestyle habits and nutrition-related information are difficult to capture and analyze in detail from a single-shot study. In addition to this, another reason might be due to the concern that overweight is determined by multiple factors so identifying a single independent risk factor might not give the best indicator.

### Limitation

The study was conducted using a cross-sectional study design which might have limited strength to explain the independent predictor for overweight among adolescents in a single shot. Likewise, change in lifestyle and overweight over time is difficult to measure. The study findings are completely based on self-reporting by the school adolescents and such findings are likely to suffer from over-or under-reporting and recall bias. Further, it is also not generalizable to the national level. Similarly, the overweight has been assessed using BMI score alone which is not considered as enough. So, the study using updated marker (waist circumference to height ratio) need to be designed in future in order to assess the adiposity among adolescents.

## Conclusion

Overweight as an emerging issue often poses a double burden among the population. Adolescents are at the greatest risk of developing behavioral and metabolic risk factors related to different kinds of non-communicable diseases. This study shows that nearly one out of ten adolescents is overweight. Overweight was found to be associated with the age of adolescents, their place of residence, birth order, maternal education, and father’s occupation. This study depicts that early-aged adolescents (11–13 years) are more overweight as compared to middle and late adolescents. In addition to this, skipping meals, type of skipped meals, consumption of green leafy vegetables and sedentary behavior has been observed as lifestyle-related predictors for overweight. However, in the final model skipping dinner was a single variable found to be statistically significant. These findings necessitate the designing of appropriate interventions targeting adolescents with specific programs focusing on adoption of healthy food habits and physical activity. Since many habits of later life begin in adolescence, concerned one need to understand the current situation of consistently prevalent under nutrition and rising over nutrition and emphasize it as a priority issue.

## Supporting information

S1 File (RAR)
